# A Rare Case of Cutaneous-Urachovesicoenteric Fistula in a Patient With Crohn’s Disease

**DOI:** 10.7759/cureus.90356

**Published:** 2025-08-18

**Authors:** Wardah Jabeen, James Clarke, Osama Shakeel, Yazid Ghanem, Nasir Z Ahmad, Prem Thomas Jacob

**Affiliations:** 1 General Surgery, University Hospital Limerick, Limerick, IRL; 2 Colorectal Surgery, University Hospital Limerick, Limerick, IRL; 3 Surgery, Cavan General Hospital, Cavan, IRL; 4 Urology, University Hospital Limerick, Limerick, IRL

**Keywords:** crohn’s disease, cutaneous, fistula, rare pathology, urachovesicoenteric fistula

## Abstract

Entero-Uracho Vesicle fistulae are a rare sequelae of aggressive Crohn’s disease, with only a small number of case reports being available in the literature. Such cases are most often managed via sepsis and skin care, nutrition, anatomy, and plan (SNAP) principles, with many Crohn’s fistulas requiring complex surgical intervention to definitively deal with the offending fistulous tracts. We present a case with an even rarer consequence of uncontrolled Crohn’s disease despite immunomodulatory therapy in a 34-year-old male who presented with an entero-uracho vesicle fistula, which was managed surgically instead of the usual SNAP protocol with excellent results.

## Introduction

Crohn’s disease is a chronic idiopathic inflammatory bowel disease process that has characteristic pathological components, including skip lesions, transmural inflammation, and the potential to affect anywhere along the alimentary canal from the mouth to the anus [1]. It can also present with a number of various extra-intestinal manifestations and is associated with a number of other immune-related disorders [2].

Following a diagnosis of Crohn’s disease, the annual incidence of hospital admission is 20% with 50% of patients needing surgical intervention within 10 years from diagnosis [3]. There is also a significantly high risk of postoperative recurrence, with approximately 44-55% patients having relapsing disease after 10 years [3].

It has been estimated that the cumulative risk of development of any type of Crohn’s fistula is 33% after 10 years and 50% after 20 years, with perianal fistulae the most common type with 21% at 10 years [4]. This leaves a significant number of patients with fistulating disease requiring surgical intervention, with many rare and varying complex presentations evident in the literature. This abstract was presented in the Grand Round Meeting at University Hospital, Limerick, in September 2024.

## Case presentation

A 34-year-old Caucasian male presented through the surgical assessment unit emergently with symptoms of lower abdominal pain with froth and debris evident on urination. He was also experiencing systemic features of fever, including sweating with rigors. Over the course of the previous four to five-month period, he had experienced significant weight loss as a consequence of uncontrolled diarrhoea, which required symptomatic treatment in the primary care. He had a background history of Crohn’s disease, diagnosed six months ago, with rapid progression despite immunotherapy with Humira and steroids use two weeks before the presentation. There was no other significant medical history of note. 

On examination, there was a palpable, hard, fixed, and tender mass of approximately 4cm x 4cm in diameter with erythema around the umbilicus, but no evidence of any skin breakage or discharge. He was also passing frothy urine.

Investigations

**Figure 1:**
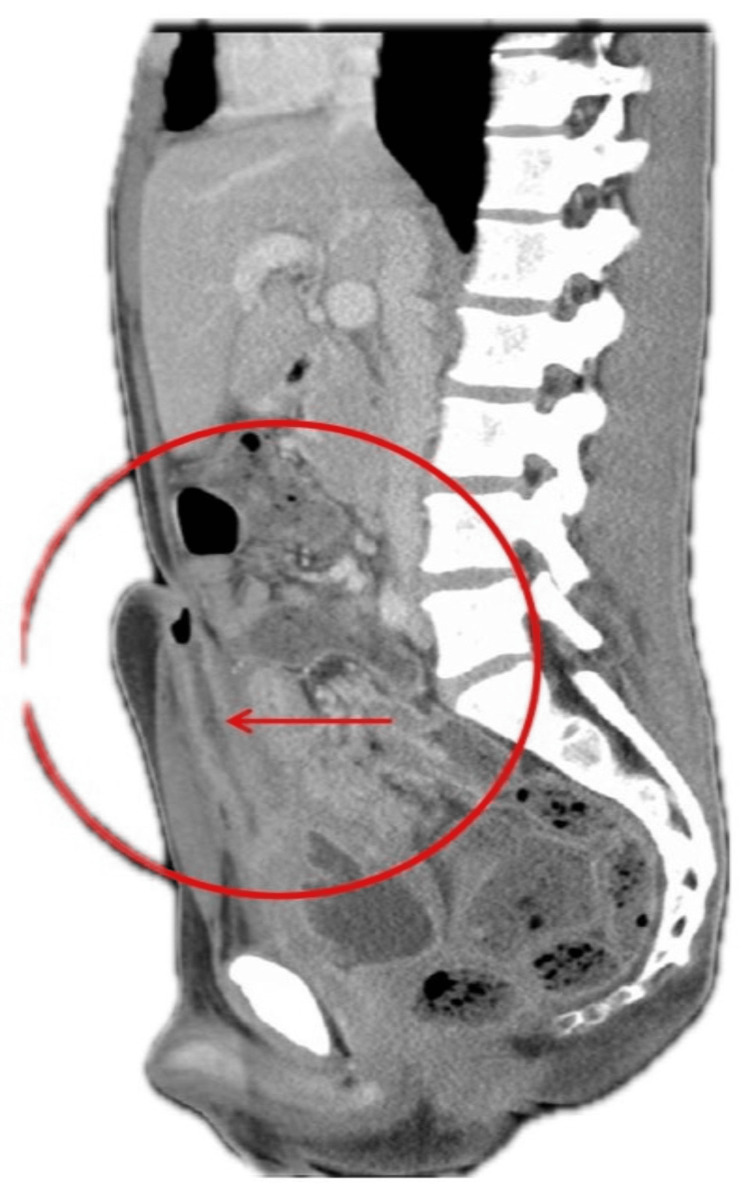
Sagital computed tomography showing fistulating tract (arrow) extending from the anterior wall of the bladder to the urachus remnant. Circle Marked inflammatory tissue of the small bowel adjacent to the superior aspect of the bladder.

Initial blood tests revealed raised inflammatory markers, including a C-reactive protein of 99 mg/L (less than 3mg/L) and albumin of 21 g/L (34 to 54 g/L). Basic haematological testing also showed a pre-existing anaemia with a haemoglobin level of 9.7 g/dL (13.5 to 18.0 g/dL). Urinalysis revealed positive leucocytes and nitrites with mixed organism growth apparent on formal mid-stream urine culture. He subsequently had computed tomography (CT) of his abdomen and pelvis, revealing significant inflammation of the anterior wall of his bladder with a fistula extending from his anterior-superior bladder into the remnant urachus tract with a highly inflamed segment of small bowel, including the ileum, in close proximity to the observed fistula. There was a persistent abscess protruding into the umbilicus. The sagittal section of the CT scan showed a fistulating tract (arrow) extending from the anterior wall of the bladder to the urachus remnant (Figure 1).

**Figure 2:**
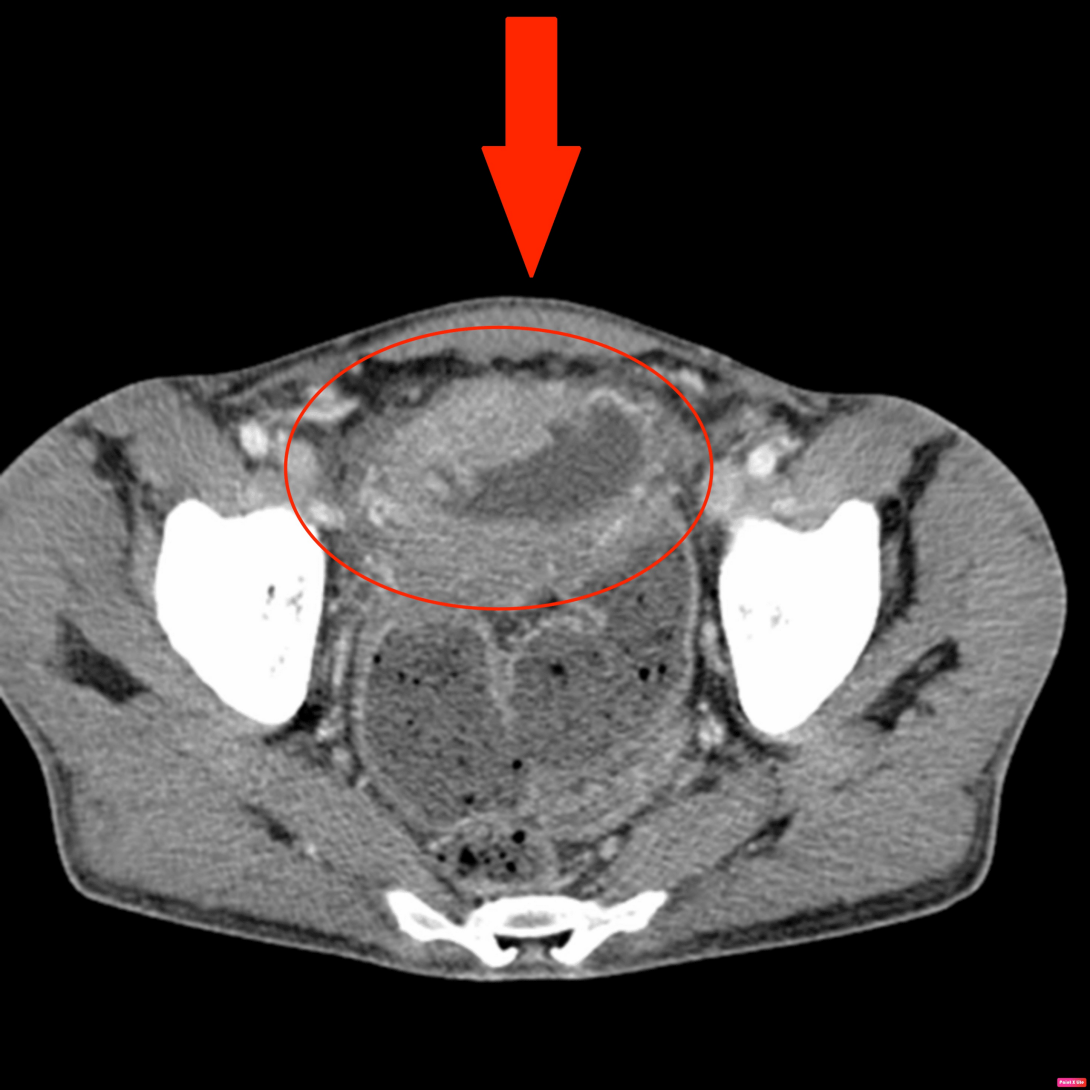
Axial computed tomography showing marked thickening and inflammation of the bladder wall.

An axial section of a CT scan showing marked thickening and inflammation of the bladder wall (Figure 2).

Treatment

Initially, he was placed on broad-spectrum antibiotic coverage, including piperacillin-tazobactam and metronidazole intravenously for an extended gram-negative and anaerobic coverage. He had a urinary catheter placed to monitor for amount of debris being discharged from the bladder. Following a prolonged period of malnutrition before presentation and with an albumin level of 21 g/L (34 to 54 g/L), the dietitian's input was sought. He was subsequently placed on a high-protein, high-calorie total parenteral nutrition (TPN) regimen. 

A multi-disciplinary approach was taken to his care with significant input from the gastroenterology, colorectal, and urology perspectives. Following a week-long period of increased nutrition, he was brought to the theatre on this index presentation. 

**Figure 3:**
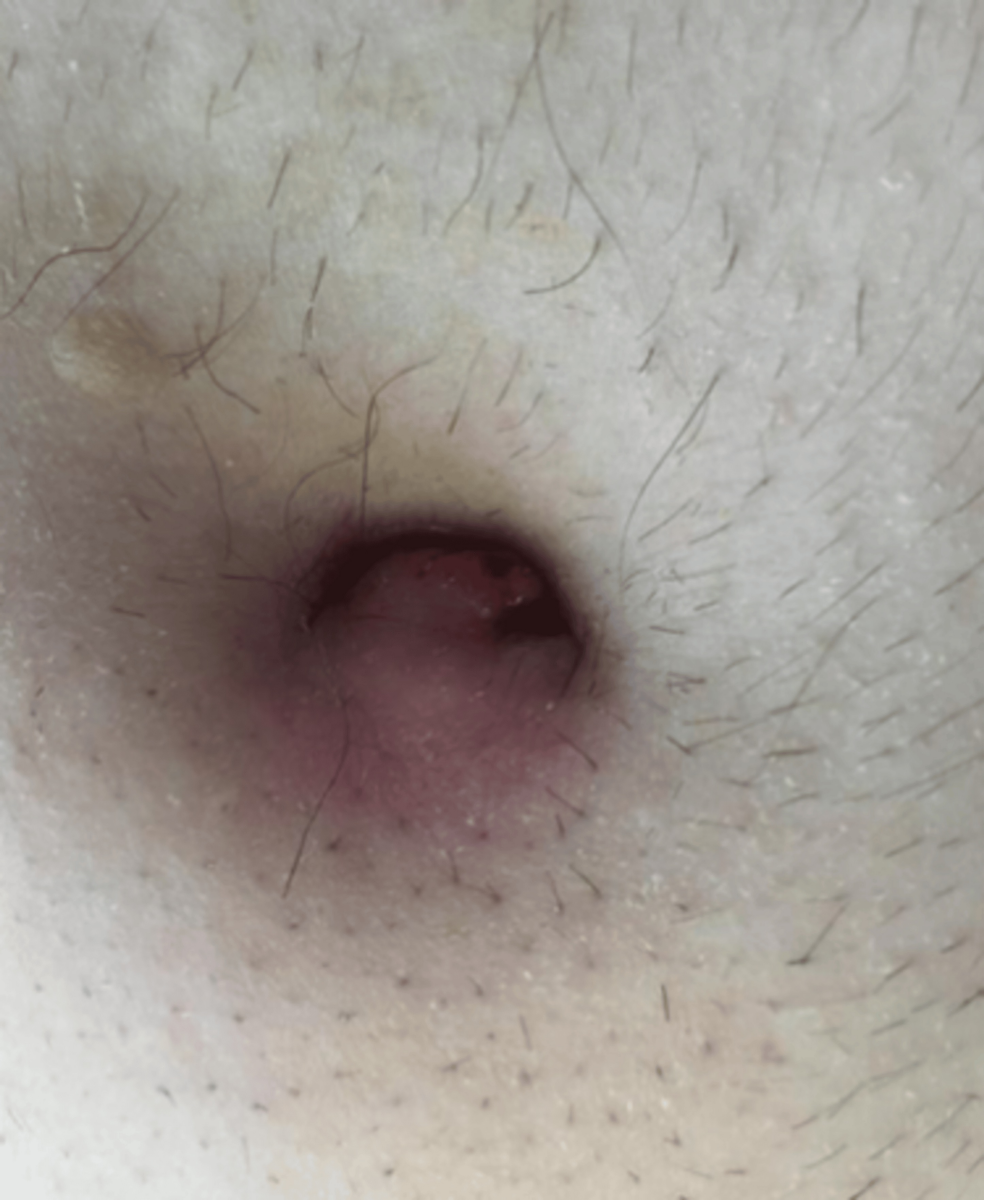
Umbilical externalization of fistula tract seen with surrounding erythema.

Of note, one day before his scheduled urgent surgical intervention, the known Entero-Uracho Vesicle fistula penetrated the skin through the umbilicus, therefore making it a cutaneous-urachovesicoenteric fistula. A stoma bag was placed over the cutaneous fistula opening to collect the discharging debris and protect the surrounding skin. Figure 3 shows the umbilical externalization of the fistula tract seen with surrounding erythema.

Intra-operatively, he initially underwent flexible cystoscopy with bilateral J stent insertion completed due to the risk of damage to the ureters in close proximity to the inflammatory mass. He subsequently had a full midline exploratory laparotomy. At exploration, there was an inflammatory mass comprising multiple loops of the distal small bowel and the wall of the bladder. A fistula was also evident from within this inflammatory mass, with connections with the anterior wall of the bladder, the ileum, and extending into the urachus remnant over a 10cm segment.

**Figure 4:**
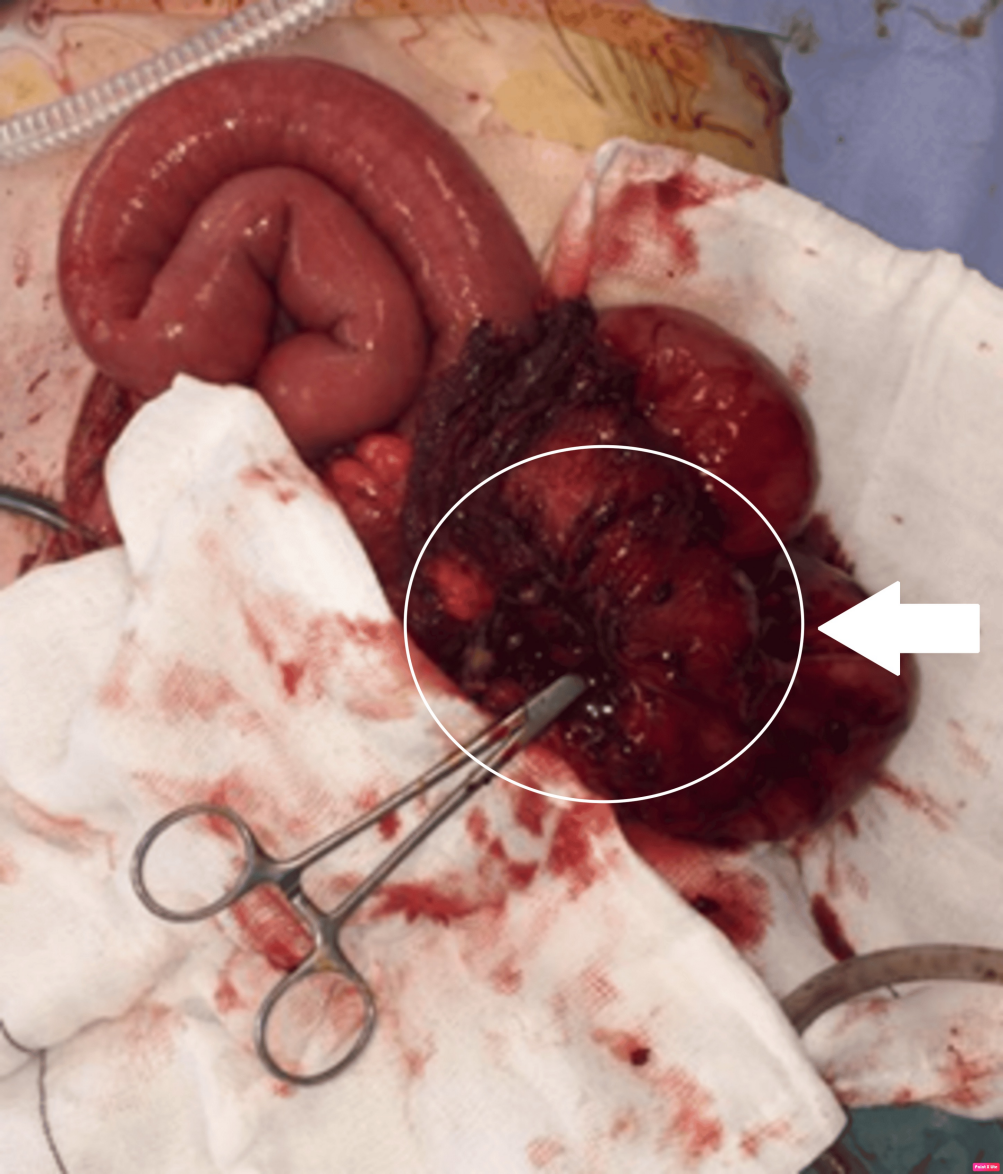
Segment of diseased small bowel with complete excision of the urachal tract with instrumentation of the fistulous opening.

The inflammatory mass was mobilised and carefully separated to definitively characterise the underlying fistula. Total excision of the fistulous tract was carried out with an ileo-caecal resection (limited right hemicolectomy) of the diseased bowel, resection of the urachus tract, and the cutaneous fistula up to the healthy margin. A primary anastomosis was successfully completed between the healthy segment of the ileum and the transverse colon. The segment of the diseased small bowel, the fistulous tract, and the urachus were all sent for histological examination. The tiny hole in the bladder was managed conservatively with a long-term urinary catheter for optimal healing, as the bladder tissues were highly inflamed and not suitable for excision. The integrity of the bladder wall was examined using sterile saline injected via the urinary catheter to ensure no leakage of intravesical contents at the end of the procedure. No defined leakage was apparent, and therefore, bladder wall resection was not carried out. A surgical non-vacuum drain was left within the pelvis to prevent any accumulation of free fluid postoperatively. Per-operative figure showing a segment of diseased small bowel with complete excision of the urachal tract with instrumentation of the fistulous opening (Figure 4).

Outcome

Post-operatively, he was transferred to the high dependency unit. On day two post presentation, he developed fevers at 38.9 °C with surgical emphysema noted on exam of the anterior abdominal wall because of extensive subcutaneous dissection. He had an emergent CT of the abdomen and pelvis, which showed significant subcutaneous emphysema but no dehiscence of the anastomosis. Subsequently, an intra-operative specimen of urine cultured Candida species, and he was therefore placed on Fluconazole with input from our local Microbiology service. 

Over the next few days, his surgical emphysema resolved spontaneously, and he progressively improved with a gradual increase in oral intake, return of bowel function, and mobility. In total, he had 14 days of intravenous antibiotic coverage post-operatively. Before discharge, he had a follow-up flexible cystoscopy with the removal of J stents and a subsequent fluoroscopic cystogram showing no evidence of loss of bladder wall integrity or leakage. 

On follow-up, histopathology results returned showing fibrous tissue of both the urachal tract and the umbilical sinus with both chronic and acute inflammatory changes evidence consistent with a fistula. Histopathological analysis of the resected bowel was consistent with Crohn’s Disease, with proximal and distal margins free of disease and no evidence of any underlying malignant process. This indicated a positive result from a surgical margin perspective. As a consequence, this result should give this patient the best chance of disease-free survival in the future. 

## Discussion

Internal fistula tracts in Crohn’s disease are well documented in the literature, as are enterocutaneous fistulas, with many fistula tracts involving the bladder, intestines, vagina, blood vessels, and skin. However, spontaneous external fistulas in the absence of prior surgical intervention are considerably rare [5, 6]. 

Previous reports in the literature have found that only 57% of spontaneous enterocutaneous fistulas responded to anti-tumour necrosis factor (TNF) therapy, while one hundred percent of spontaneous fistulas closed after surgery [7]. This is in line with our case, wherein the fistula tract continued to progress and worsen while on anti-TNF therapy, eventually fistulising with the urachal tract and umbilicus. 

From our search of the major literature databases, umbilical fistulisation was found to be relatively prevalent in the literature, but umbilical fistulisation with enteric, urachal, and vesical involvement has only been reported once before by Eugene D Davidson in 1980, who reported a Crohn’s patient with spontaneous cutaneous-urachovesicoenteric fistula [8]. 

In their case, Davidson reported a 28-year-old male patient with previously undiagnosed Crohn’s disease who presented with gastrointestinal symptoms and an ulcerated lesion in the umbilicus that drained pus and food particles. Sigmoidoscopy was performed, and an intravenous pyelogram with retrograde pyelogram and cystogram was normal. A fistula tract was demonstrated in the anterior right dome of the bladder on cystoscopy, and contrast injected through the umbilical sinus travelled into the small bowel, bladder, and cecum. Open abdominal exploration revealed a persistent urachal tract leading to an inflammatory tract in the right lower quadrant, involving the bladder, terminal ileum, and cecum. The patient was managed by resecting the entire area, including a 2.5-foot length of terminal ileum and cecum. Primary anastomosis with bladder wall repair was performed, and the patient had an uneventful recovery. 

Unlike the case reported by Davidson, our case’s presentation was mainly due to abdominal pain with a pronounced vesical element, with evident urinary debris and frothy urine. This then developed into a cutaneous infra-umbilical fistula. At that time, the patient was already nil per oral (NPO) and was on total parenteral nutrition, thus minimal faecal discharge was seen from the external fistula tract. Furthermore, our case did not require endoscopy confirmation as the patient had a previously confirmed and documented diagnosis of Crohn’s disease. Computed Tomography was sufficient to clearly visualize the fistula tract and aid in surgical planning.

Additionally, our patient received bilateral J stents to address the risk of intraoperative ureteric injury due to their proximity to the fistula tract. While Davidson did not take this approach in the case reported by him, the patient still had good outcomes. The ureter is a retroperitoneal structure, and on our dissection, it was far away from the operating field; however, for the sake of intraoperative safety, a ureteric stent was in situ.

Finally, total excision of the fistulous tract was carried out with an ileo-caecal resection of the diseased bowel, resection of the urachus, and closure of the cutaneous fistula site. Primary anastomosis was performed with a desirable outcome. This is in a similar fashion to the case reported by Davidson.

Davidson emphasizes the importance of a full and extensive diagnostic workup, which at the time included intravenous pyelogram (IVP), cystogram, cystoscopy, upper GI series, small bowel series, and barium enema, as well as a sonogram from the umbilical opening. Since then, these procedures have been phased out in favour of radiological imaging owing to the advancement of radiological equipment and software, with our case being diagnosed primarily with computed tomography. Cystoscopy would still be of value to visualize the fistula tract and prophylactically insert J stents, as such complicated fistulae require extensive resection in a sensitive area, risking injury to delicate structures such as the ureter.

In his case, Davidson also points out the importance of preoperative nutritional support, which our patient received through total parenteral nutrition, and continued postoperatively while oral feeding was introduced until he could meet his nutritional requirements. 

It is vital to involve multidisciplinary care in the management of such patients, as they will present with multiple issues requiring detailed solutions. Our patient required input from gastroenterology, urology, colorectal surgery, microbiology, dietetics, and physiotherapy, among others, to achieve full recovery.

Of note, this patient suffered from urinary candidiasis, the origin of which has not been determined. Šašala et al. demonstrated that patients with Crohn’s disease had a higher prevalence of fungi and yeasts in the abdomen compared to a cancer patient group undergoing abdominal surgery [9]. A non-causal relationship between Crohn’s fistula formation or Crohn’s pathogenesis and abdominal fungal colonisation could be an interesting research topic to consider in the future. 

## Conclusions

Crohn’s Disease can present in many forms of clinical manifestations. A significant proportion will develop severe disease with subsequent fistula formation, the most common of which is perianal fistula. However, there is a wide variety of potential internal fistulae that can develop from this systemic condition, as seen in this rare presentation. These are commonly managed via a strict adherence to sepsis control, skin care, nutritional support, defined anatomy, and develop surgical procedure (SSNAP). Surgical intervention is required in up to 50% of patients within 10 years of the time of initial Crohn’s diagnosis. Many such cases are due to fistulating disease. However, due to the nature of this condition, lifelong vigilance is necessary due to the high risk of relapsing disease.
